# Divergent Synthesis of Quinolone Natural Products from *Pseudonocardia* sp. CL38489

**DOI:** 10.1002/ejoc.201601195

**Published:** 2016-11-15

**Authors:** Stephen M. Geddis, Laura Carro, James T. Hodgkinson, David R. Spring

**Affiliations:** ^1^ Department of ChemistryUniversity of CambridgeLensfield RoadCB2 1EWCambridgeUK

**Keywords:** Quinolones, Natural products, Antibacterial agents, Cross‐coupling, Michael addition

## Abstract

Two divergent synthetic routes are reported offering access to four quinolone natural products from *Pseudonocardia* sp. CL38489. Key steps to the natural products involved a regioselective epoxidation, an intramolecular Buchwald–Hartwig amination and a final acid‐catalysed 1,3‐allylic‐alcohol rearrangement to give two of the natural products in one step. This study completes the synthesis of all eight antibacterial quinolone natural products reported in the family. In addition, this modular strategy enables an improved synthesis towards two natural products previously reported.

## Introduction

Quinolones, both natural and synthetic, have repeatedly distinguished themselves as molecules of high biological relevance, which display a plethora of activities. The synthetic fluoroquinolone antibiotics have seen widespread use, with over 800 million patients treated worldwide.^1^ These antibiotic properties have also been utilised by nature: *Pseudomonas aeruginosa* produces a number of quinolone *N*‐oxides, termed Pyo compounds, which are strongly active against gram‐positive bacteria.^2^, ^3^ It is believed that this enables *P. aeruginosa* to outcompete *Staphylococcus aureus* in cystic‐fibrosis lung infections.^4^

An area of increasing interest, in which quinolones have been implicated, is that of quorum sensing, whereby bacteria use signaling molecules to modulate their activity in a population‐density‐dependent manner. *P. aeruginosa* is known to use quinolones as such signaling molecules,^5^ and it has recently emerged that quinolones are also produced by *Buckerholdia* and *Altermonas* spp., which raises the intriguing possibility of interspecies signaling.^6^, ^7^, ^8^

**Figure 1:**
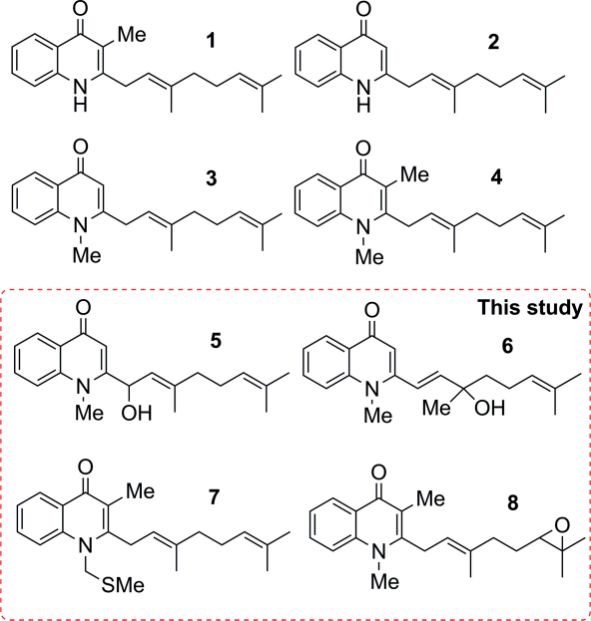
Eight quinolone natural products isolated from *Pseudonocardia* sp. CL38489.

Given this wealth of activities, we became interested in a group of quinolone natural products produced by the actinomycete *Pseudonocardia* sp. CL38489, which was originally isolated by Dekker et al. from an Indian soil sample.^9^ (Figure 1). The molecules were originally noted for their antibacterial activity against *Helicobacter pylori*, which is implicated in the pathogenesis of chronic gastritis, peptic ulcers and gastric cancers.^10^, ^11^

**Scheme 1:**
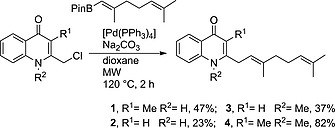
Previously employed sp^2^–sp^3^ Suzuki–Miyaura coupling to give natural products **1**–**4**.^12^

We recently described the synthesis of compounds **1**–**4**.^12^ The key step was an sp^2^–sp^3^ Suzuki–Miyaura reaction, whereby the various requisite quinolone cores were coupled with a common boronate ester (Scheme 1). However, one drawback of this modular strategy was the necessity to separately synthesise each quinolone coupling partner, with up to seven steps required for each quinolone core.

**Scheme 2:**
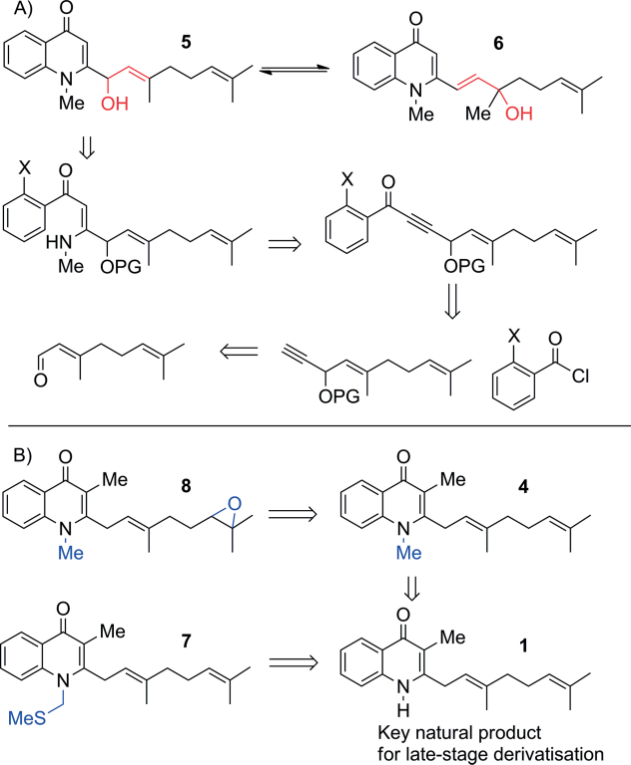
(A) Anticipated equilibration between **5** and **6** could enable access to both products from one synthetic scheme; retrosynthesis of natural product **5**. X = halogen, PG = protecting group. (B) Retrosynthesis of natural‐product quinolones **8**, **4** and **7** from key natural product **1**.

In this study, our attention turned towards the synthesis of the remaining four natural products. While devising the strategy towards these, the similarity between them was noted. It was envisaged that this would enable a divergent approach towards the natural products, with decoration of mutual late‐stage intermediates leading to multiple products through the same synthetic scheme. This approach would offer a much improved efficiency over the original routes.^13^ In particular, the isomeric relationship between **5** and **6** was noted. It was anticipated that under acidic conditions, the allylic alcohol moiety in **5** could potentially undergo a 1,3‐rearrangement to give **6**, which allows interconversion between the two molecules (Scheme 2A). We hypothesised that the additional conjugated system in **6** would be the driving force for the rearrangement. However, due to the differences in the lateral chain of **5** and **6** compared to the rest of the natural products, a retrosynthetic strategy alternative to the sp^2^–sp^3^ Suzuki–Miyaura route was required. For natural products **7** and **8** it was noted that the natural products contained previously synthesised **1** as the core structure, which could act as an intermediate in the synthesis of both (Scheme 2B). The synthesis of **8** would also require natural product **4** as an intermediate. The proposed strategy would therefore enable the synthesis of a total of six natural products from just two synthetic routes.

## Results and Discussion

The synthesis of **5** took its inspiration from the method of Bernini et al., in which the 4‐quinolone core is assembled by means of a copper‐catalysed heterocyclisation of 1‐(2‐halophenyl)‐2‐en‐3‐amin‐1‐ones, which are in turn synthesised from α,β‐ynones and primary amines.^14^ Several similar methodologies exist;^15^, ^16^ however, the use of these to attain 1,2‐dialkyl‐4‐quinolones has been limited thus far.

**Scheme 3:**
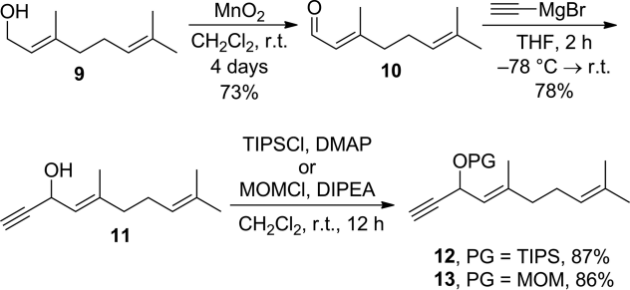
Synthesis of protected propargylic alcohol coupling partners **12** and **13** (DMAP = 4‐dimethylaminopyridine; DIPEA = *N*,*N*‐diisopropylethylamine).

The requisite alkyne commenced with the already reported oxidation of geraniol **9** to geranial **10**,^17^ followed by the reaction with ethynyl magnesium bromide to give propargylic alcohol **11** (Scheme 3). It was then necessary to protect the hydroxyl group to preclude the competing esterification in the subsequent Sonogashira‐coupling step; triisopropylsilyl (TIPS) and methoxymethyl ether (MOM) protection proceeded smoothly with good yields to give **12** and **13**.

**Scheme 4:**
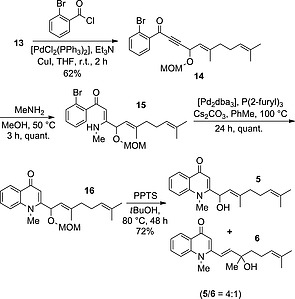
Synthesis of natural products **5** and **6**. Attempted deprotection of **16** also resulted in a partial 1,3‐allylic‐alcohol rearrangement, which allowed both to be isolated (PPTS = pyridinium *p*‐toluenesulfonate).

Compounds **12** and **13** were then subjected to Bernini's Sonogashira conditions (Scheme 4).^14^ Compound **12** gave no reaction, presumably due to the presence of the bulky silyl group; however, **13** gave α,β‐ynone **14** in a moderate yield. This ynone was then subjected to a Michael addition with methylamine, which proceeded quantitatively to give **15**. A number of conditions were then trialled to elicit the key heterocyclisation. Notably, the copper‐catalysed conditions by Bernini et al. gave no product, as monitored by liquid chromatography–mass spectrometry (LCMS), but instead led to the formation of a dimer. However, Buchwald–Hartwig conditions^18^ brought about the required transformation to give **16** in a quantitative yield, which then merely required deprotection to give **5**. To our delight, when this was attempted by using pyridinium tosylate, **6** was also produced, which indicated that the predicted 1,3‐allylic‐alcohol rearrangement was taking place. The two compounds could be separated by preparative HPLC, which gave access to both natural products. We speculate that the reaction proceeds through elimination of water to give an allylic cation, which reacts with water to give either **5** or **6** depending on the position of attack. The fact that both products were originally isolated in optically enriched form may perhaps imply the existence of an analogous enzymatic process, because our proposed mechanism would result in racemisation.

**Scheme 5:**
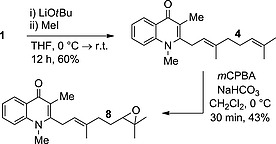
Synthesis of natural products **4** and **8** directly from **1** (*m*CPBA = *meta*‐chloroperoxybenzoic acid).

With these natural products in hand, attention was then turned to **7** and **8**. The proposed strategy required natural product **1** as point of divergence; it was prepared according to our previously published procedure.^12^ Natural product **1** was then subjected to methylation conditions by using LiO*t*Bu as a base (Scheme 5); the choice of base is important to ensure selectivity for *N*‐ versus *O*‐methylation.^19^ This gave natural product **4** in a moderate yield, which then underwent regioselective epoxidation to give **8**. The production of three natural products in direct succession demonstrates the high efficiency of this divergent strategy.

**Scheme 6:**
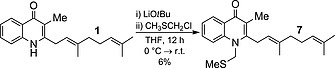
Low‐yielding conversion of **1** to **7**.

**Scheme 7:**
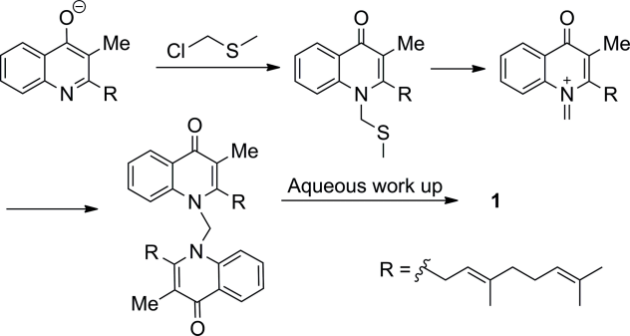
Proposed competing dimerisation accounting for low conversion of **1** to **7**.

The methylthiomethylenation of **1** to give **7** was then attempted. Analogous conditions to those used in the methylation of **1** were trialled to give **7** with a very poor yield (6 %); starting material was also recovered (37 %; Scheme 6). Optimisation was attempted with a variety of bases (NaH, LiH, *n*BuLi), solvents (DMF, THF, DMSO), temperatures and concentrations, none of which offered any improvement. TLC analysis of these reactions revealed that a non‐polar species was present, which appeared to gradually decompose to starting material. This prompts us to postulate that a competing elimination reaction leads induces the formation of a dimer, which hydrolyses to starting material upon workup and hence precludes high reaction conversion (Scheme 7; similar dimers have been isolated in the literature, but this was not possible in our case).^20^ Additionally, a pure sample of **7** was observed to be stable with respect to the work‐up conditions, which implied that the low yield is not due to product decomposition following the quench. Nonetheless, a sufficient amount of **7** was isolated to facilitate future biological testing.

## Conclusions

Two synthetic routes were developed, offering divergent access to six natural products, four of which (**5**–**8**) have never before been synthesised. The yields were moderate to excellent, with one exception; however, the main benefit of the divergent strategy is the low number of steps required to yield multiple targets. Considering the essential need for new antibacterial agents, such compounds could play a crucial role in discovering novel modes of action and act as new leads. Following biological screening, the routes will also be suitable for rapid generation of analogues, which will enable the investigation of structure–activity relationships of any activities detected.

## Experimental Section

**Supporting Information** (see footnote on the first page of this article): Full experimental protocols, characterisation data and ^1^H and ^13^C NMR spectra.

## Supporting information

Supporting Information
