# Supplementary material for: Divergent Synthesis of Quinolone Natural Products from Pseudonocardia sp. CL38489
Source: European J Org Chem. 2016 Nov 15;2016(35):5799–802. doi: 10.1002/ejoc.201601195 (PMC5215369; doi:10.1002/ejoc.201601195)

Eur. J. Org. Chem. **2016** · ISSN 1099–0690

SUPPORTING INFORMATION

DOI: 10.1002/ejoc.201601195
Title: Divergent Synthesis of Quinolone Natural Products from *Pseudonocardia* sp. CL38489
Author(s): Stephen M. Geddis, Laura Carro, James T. Hodgkinson, David R. Spring*

General Experimental 2
(*E*)-3,7-dimethylocta-2,6-dienal (10) 2
(*E*)-5,9-dimethyldeca-4,8-dien-1-yn-3-ol (11) 3
(*E*)-((5,9-dimethyldeca-4,8-dien-1-yn-3-yl)oxy)triisopropylsilane (12) 3
(*E*)-3-(methoxymethoxy)-5,9-dimethyldeca-4,8-dien-1-yne (13) 4
(*E*)-1-(2-bromophenyl)-4-(methoxymethoxy)-6,10-dimethylundeca-5,9-dien-2-yn-1-one (14) 4
(2*Z*,5*E*)-1-(2-bromophenyl)-4-(methoxymethoxy)-6,10-dimethyl-3-(methylamino)undeca-2,5,9-trien-1-one (15) 5
(*E*)-2-(1-(methoxymethoxy)-3,7-dimethylocta-2,6-dien-1-yl)-1-methylquinolin-4(1*H*)-one (16) 6
(*E*)-2-(1-hydroxy-3,7-dimethylocta-2,6-dien-1-yl)-1-methylquinolin-4(1*H*)-one (5) & (*E*)-2-(3-hydroxy-3,7-dimethylocta-1,6-dien-1-yl)-1-methylquinolin-4(1*H*)-one (6) 6
(*E*)-2-(3,7-dimethylocta-2,6-dien-1-yl)-3-methylquinolin-4(1*H*)-one (1) 7
(*E*)-2-(3,7-dimethylocta-2,6-dien-1-yl)-1,3-dimethylquinolin-4(1*H*)-one (4) 8
(*E*)-2-(5-(3,3-dimethyloxiran-2-yl)-3-methylpent-2-en-1-yl)-1,3-dimethylquinolin-4(1*H*)-one (8) 9
(*E*)-2-(3,7-dimethylocta-2,6-dien-1-yl)-3-methyl-1-((methylthio)methyl)quinolin-4(1*H*)-one (7) 10
References 10
^{1}H and ^{13}C spectra 11

General Experimental

^{1}H and ^{13}C nuclear magnetic resonance (NMR) spectra were recorded using an internal deuterium lock at ambient probe temperature on a Bruker DPX 400 (400 MHz), Bruker Avance 400 QNP Ultrashield (400 MHz), Bruker Avance 500 BB ATM (500 MHz) or Bruker Avance 500 Dual Cryo (500 MHz) spectrometer. Chemical shifts (δ) are quoted in ppm relative to the residual non-deuterated solvent signal. Data are reported as follows: chemical shift, integration, multiplicity (s, singlet; d, doublet; t, triplet; q, quartet; br, broad; app, apparent; or a combination of these, e.g. br s or dd), coupling constants (*J* in Hz), and assignment. The numbering system used in the assignments does not necessarily follow the IUPAC convention. Assignment of the spectra is supported by DEPT-135 or COSY, HSQC, HMBC and NOESY experiments where necessary. NMR spectra were acquired at 300 K unless otherwise indicated. All coupling constants are reported to the nearest 0.5 Hz. Infra-red (IR) spectra were recorded neat on a Perkin Elmer Spectrum One FT-IR spectrophotometer fitted with an attenuated total reflectance (ATR) sampling accessory. Selected absorption maxima (ν max) are reported in wavenumbers (cm^{-1}). Melting points were measured using a Büchi B545 meltingpoint apparatus and are uncorrected. High resolution mass spectrometry (HRMS) was carried out using a Waters LCT Premier Time of Flight (ToF) mass spectrometer or Micromass Quadrapole-Time of Flight (Q-ToF) mass spectrometer. Reported mass values are within the error limits of ±5 ppm. Analytical HPLC was run on an Agilent 1260 Infinity using a Supelcosil ABZ+PLUS column (150 mm × 4.6 mm, 3 μm) eluting with a linear gradient system (solvent A: water, solvent B: acetonitrile) over 15 min at a flow rate of 1 mL/min. Semi-preparative HPLC was run on an Agilent 1260 Infinity using a Supelcosil ABZ+PLUS column (250 mm × 21.2 mm, 5 μm) eluting with a linear gradient system (solvent A: water, solvent B: acetonitrile) over 20 min at a flow rate of 20 mL/min. HPLC was monitored by UV absorbance at 220 and 254 nm. Analytical HPLC was run on an Agilent 1260 Infinity using a Supelcosil ABZ+PLUS column (150 mm × 4.6 mm, 3 μm) eluting with a linear gradient system (solvent A: 0.05% (v/v) TFA in water, solvent B: 0.05% (v/v) TFA in acetonitrile) over 15 min at a flow rate of 1 mL/min.

(*E*)-3,7-dimethylocta-2,6-dienal (10)

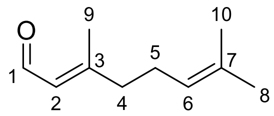

Following the procedure of Lautens et al.[1] A slurry of geraniol (3.00 mL, 17.1 mmol) and MnO_2 (85% purity, 17.5 g, 171 mmol) in CH_2Cl_2 (15 mL) was stirred at RT for 48 h, then filtered through celite, rinsing with CH_2Cl_2. The filtrate was dried ($MgSO_4$) and the solvent was removed *in vacuo*. The crude product was purified by flash column chromatography (SiO_2, 10% Et_2O, Pet. Ether 40-60) to give 5-iodo-2-methylpent-2-ene as a colourless oil (1.89 g, 12.4 mmol, 73%).

^{1}H NMR (500 MHz, $CDCl_3$): δ = 10.00 (1H, d, *J* = 8.2 Hz, H-1), 5.90-5.87 (1H, m, H-2), 5.09-5.05 (1H, m, H-6), 2.26-2.19 (2H, m, H-4 & H-5), 2.17 (3H, s, H-9), 1.69 (3H, s, H-8), 1.61 (3H, s, H-10); **^{13}C NMR** (125 MHz, $CDCl_3$): δ = 191.3 (C-1), 163.9 (C-3), 132.9 (C-7), 127.4 (C-7), 122.5 (C-6), 40.6 (C-4), 25.7 (C-8 or C-5), 25.6 (C-8 or C-5), 17.7 (C-9 or C-10), 17.6 (C-9 or C-10); ***ν*** [cm^{-1}] (neat): 1671 (s, C=O aldehyde); analytical data consistent with that previously published.[1]

(*E*)-5,9-dimethyldeca-4,8-dien-1-yn-3-ol (11)

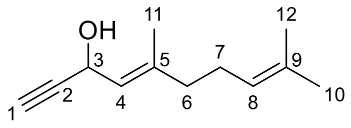

To a solution of **10** (1.23 g, 8.08 mmol) in THF (40 mL) at -78 °C was added dropwise ethynylmagnesium bromide (0.5 M in THF, 32.4 mL, 16.2 mmol). The mixture was allowed to return to RT, then stirred for 2 h. Sat. aq. NH_4Cl (20 mL) was added dropwise, followed by extraction with EtOAc (3 x 20 mL). The combined organic extracts were washed with brine (30 mL), dried ($MgSO_4$), then concentrated *in vacuo*. The crude product was purified by flash column chromatography (SiO_2, 20% Et_2O/ pet. ether 40-60) to give the title compound as a yellow oil (1.18g, 6.30 mmol, 78%)

^{1}H NMR (500 MHz, $CDCl_3$): δ = 5.42-5.39 (1H, m, H-4), 5.13-5.09 (2H, m, H-3 & H-8), 2.52 (1H, d, *J* = 2.1 Hz, H-1), 2.17-2.11 (2H, m, H-7), 2.08-2.05 (2H, m, H-6), 1.08 (1H, d, *J* = 5.2 Hz, O-H), 1.71 (3H, d, *J* = 1.2 Hz, H-11), 1.63 (3H, s, H-10), 1.60 (3H, s, H-12); **^{13}C NMR** (125 MHz, $CDCl_3$): δ = 141.1 (C-5), 132.0 (C-9), 124.0 (C-4), 123.6 (C-8), 84.5 (C-2), 72.5 (C-1), 59.0 (C-3), 39.3 (C-6), 26.2 (C-7), 25.7 (C-10), 17.7 (C-12), 16.6 (C-11); ***ν*** [cm^{-1}] (neat): 3300 (m br, O-H), 3286 (m, C≡C-H), 1666 (w, C=C); **HRMS**: *m/z* (ESI) calcd for $C_{12}H_{19}O$ $[M+H]^+$: 179.1430, found 179.1426; **R*f*** (10% Et_2O/ pet. ether 40-60) = 0.11

(*E*)-((5,9-dimethyldeca-4,8-dien-1-yn-3-yl)oxy)triisopropylsilane (12)

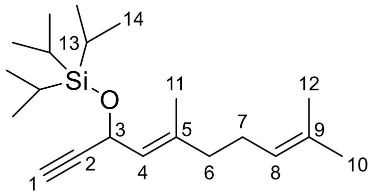

To a solution of **11** (100 mg, 0.53 mmol) and DMAP (129 mg, 1.06 mmol) in CH_2Cl_2 (3 mL) was added TIPSCl (204 mg, 1.06 mmol), followed by stirring for 90 h. Sat. aq. $NaHCO_3$ (3 mL) was added, the phases separated, and the aqueous phase extracted with Et_2O (3 x 2 mL). The combined organic phases were washed with (3 mL), brine (3 mL), dried (Na_2SO_4) and concentrated *in vacuo*. The crude product was purified by flash column chromatography (SiO_2, 5% Et_2O/ pet. ether 40-60) to give the title compound as a colourless oil (69.2 mg, 0.31 mmol, 58%)

^{1}H NMR (500 MHz, $CDCl_3$): δ = 5.39-5.36 (1H, m, H-4), 5.15 (1H, dd, J = 7.9, 2.1 Hz, H-3), 5.13-5.09 (1H, m, H-8), 2.45 (1H, d, J = 2.4 Hz, H-1), 2.16-2.10 (2H, m, H-7), 2.07-2.03 (3H, m, H-6), 1.70-1.69 (6H, m, H-10 & H-11), 1.62 (3H, s, H-12), 1.16-1.07 (21H, m, H-13 & H-14); **^{13}C NMR** (125 MHz, $CDCl_3$): δ = 136.7 (C-5), 131.7 (C-9), 126.1 (C-4), 123.8 (C-8), 85.1 (C-2), 71.4 (C-1), 60.0 (C-3), 39.1 (C-6), 26.1 (C-7), 25.7 (C-10), 17.9 (C-12 or C-14), 17.8 (C-12 or C-14), 16.6 (C-11), 12.2 (C-13); ***ν*** [cm^{-1}] (neat): 3311 (w, C≡C-H), 2361 (w, C≡C); **HRMS:** *m/z* (ASAP) calcd for $C_{21}H_{39}OSi$ $[M+H]^+$: 335.2770, found 335.2766; **R*f*** (10 % Et_2O/ pet. ether 40-60) = 0.87

(*E*)-3-(methoxymethoxy)-5,9-dimethyldeca-4,8-dien-1-yne (13)

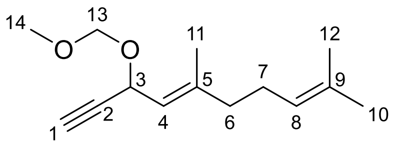

To a solution of **11** (1.00 g, 5.61 mmol) and DIPEA (2.9 mL, 16.8 mmol) in CH_2Cl_2 (30 mL) was added MOMCl (0.85 mL, 11.22 mmol), followed by stirring at RT for 48 h. Sat. aq. $NaHCO_3$ (10 mL) was added. The aqueous phase was extracted with Et_2O (3 x 20 mL), then the combined organic phases were washed with brine (30 mL), dried (Na_2SO_4) then concentrated *in vacuo*. The crude product was purified by flash column chromatography (4% Et_2O/ pet. ether 40-60) to give the title compound as a colourless oil (1.07 g, 4.81 mmol, 86%)

^{1}H NMR (500 MHz, $CDCl_3$): δ = 5.34-5.31 (1H, m, H-4), 5.11-5.07 (2H, m, H-3 & H-8), 4.85 (1H, d, J = 7.0 Hz, H-13^a), 4.63 (1H, d, J = 6.7 Hz, H-13^b), 3.41 (3H, s, H-14), 2.47 (1H, d, J = 2.1 Hz, H-1), 2.17- 2.11 (2H, m, H-6), 2.09-2.05 (2H, m H-7), 1.74 (3H, d, J = 1.2 Hz, H-11), 1.69 (3H, s, H-10), 1.61 (3H, s, H-12); **^{13}C NMR** (125 MHz, $CDCl_3$): δ = 141.8 (C-5), 131.9 (C-9), 123.6 (C-8), 121.5 (C-4), 93.1 (C-12), 82.2 (C-2), 73.1 (C-1). 61.5 (C-3), 55.6 (C-14), 39.3 (C-6), 26.2 (C-7), 25.7 (C-10), 17.7 (C-12), 16.6 (C-11); ***ν*** [cm^{-1}] (neat): 3285 (w, C≡C-H), 2352 (w, C≡C); HRMS could not be obtained; **R*f*** (10 % Et_2O/ pet. ether 40-60) = 0.50

(*E*)-1-(2-bromophenyl)-4-(methoxymethoxy)-6,10-dimethylundeca-5,9-dien-2-yn-1-one (14)

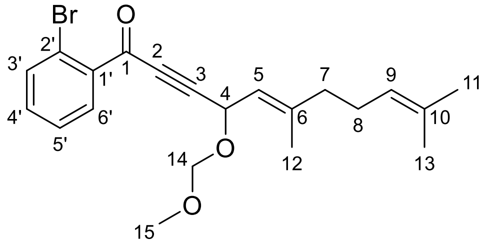

A mixture of 2-bromobenzyl chloride (0.15 mL, 1.11 mmol), $PdCl_2(PPh_3)_2$ (16 mg, 0.92 mmol) and Et_3N (0.15 mL, 1.11 mmol) and THF (6 mL) was stirred at RT for 10 m. CuI (7 mg, 0.033 mmol) was

added, followed by a further 10 m of stirring at RT. **13** (0.21 mL, 0.92 mmol) was added, followed by stirring for 1 h 45 m. The mixture was then diluted with EtOAc (15 mL), washed with sat. aq. NH_4Cl (10 mL), dried (Na_2SO_4) and concentrated *in vacuo*. The crude product was purified by flash column chromatography (SiO_2, 40-60% CH_2Cl_2/ pet. ether 40-60) to give the title compound as an orange oil (233 mg, 0.57 mmol, 62%).

^{1}H NMR (500 MHz, $CDCl_3$): δ = 8.03 (1H, dd, J = 7.6, 1.8 Hz, H-6'), 7.70 (1H, dd, J = 7.9, 1.2 Hz, H-3'), 7.45 (1H, td, J = 7.6, 1.2 Hz, H-5'), 7.39 Hz (1H, td, J = 7.6, 1.8 Hz, H-4'), 5.39-5.36 (1H, m, H-5) 5.32 (1H, d, J = 8.9 Hz, H-4), 5.12-5.08 (1H, m, H-9), 4.87 (1H, d, J = 7.0 Hz, H-14^a), 4.69 (1H, d, J = 6.7 Hz, H-14^a), 3.43 (3H, s, H-15), 2.18-2.09 (4H, m, H-7 & H-8), 1.79 (3H, s, H-12), 1.69 (3H, s, H-11), 1.62 (3H, s, H-13); **^{13}C NMR** (125 MHz, $CDCl_3$): δ = 176.9 (C-1), 143.3 (C-5), 136.9 (C-11), 135.0 (C-3'), 133.4 (C-4'), 133.1 (C-6'), 132.1 (C-10), 127.3 (C-5'), 123.4 (C-9), 121.2 (C-2'), 119.7 (C-5), 93.6 (C-16), 93.2 (C-3), 83.4 (C-2), 61.9 (C-4), 55.8 (C-15), 39.4 (C-7), 26.1 (C-8), 25.7 (C-11), 17.7 (C-13), 16.8 (C-12); ***ν*** [cm^{-1}] (neat): 2206 (C≡C), 1655 (s, C=O ketone), 1586 (w, C=C Ar), 1563 (w, C=C Ar); **HRMS:** *m/z* (ASAP) calcd for $C_{21}H_{26}O_3Br$ [M+H]$^+$: 405.1065, found 405.1054; **R*f*** (40 % CH_2Cl_2 / pet. ether 40-60) = 0.14

(2*Z*,5*E*)-1-(2-bromophenyl)-4-(methoxymethoxy)-6,10-dimethyl-3-(methylamino)undeca-2,5,9-trien-1-one (15)

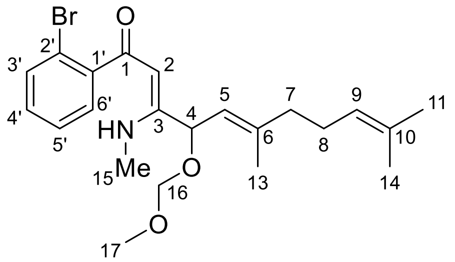

To a solution **14** (233 mg, 0.57 mmol) in MeOH (1.8 mL) was added $MeNH_2$ (2.0 M in MeOH, 0.57 mL, 1.15 mmol). The reaction vessel was sealed, followed by heating and stirring at 50 °C for 3 h. The solution was cooled and concentrated *in vacuo* to give the title compound as an orange oil (248 mg, 0.57 mmol, 100%).

^{1}H NMR (500 MHz, $CDCl_3$): δ = 11.05 (1H, d, J = 4.8 Hz, N-H), 7.57 (1H, dd, J = 7.9, 0.9 Hz, H-6'), 7.44 (1H, dd, J = 7.6, 1.5 Hz, H-3'), 7.31 (1H, td, J = 7.5, 1.2 Hz, H-4'), 7.20-7.17 (1H, m, H-5'), 5.65 (1H, s, H-2), 5.24-5.21 (1H, m, H-5), 5.10 (1H, d, J = 9.16 Hz, H-5), 5.08-5.05 (1H, m, H-9), 4.65 (3H, s, H-16), 3.40 (3H, s, H-17), 3.03 (3H, d, J = 5.5 Hz, H-15), 2.17-2.11 (4H, m, H-7 & H-8), 1.82 (3H, d, J = 1.5 Hz, H-13), 1.68 (3H, s, H-11), 1.61 (3H, s, H-14); **^{13}C NMR** (125 MHz, $CDCl_3$): δ = 190.8 (C-1), 167.7 (C-3), 143.9 (C-6), 143.5 (C-1'), 133.2 (C-6'), 132.1 (C-10), 129.9 (C-5'), 129.1 (C-3'), 127.1 (C-4'), 123.4 (C-9), 120.4 (C-5), 119.5 (C-2'), 93.5 (C-16), 92.7 (C-2), 69.2 (C-4), 55.8 (C-17), 39.6 (C-7), 29.8 (C-15), 26.2 (C-8), 25.7 (C-11), 17.7 (C-14), 16.8 (C-13); ***ν*** [cm^{-1}] (neat): 2910 (m, N-

H), 1605 (s, C=O ketone), 1570 (s, C=C Ar); **HRMS**: *m/z* (ES) calcd for $C_{22}H_{31}NO_3$ $[M+H]^+$: 436.1487, found 436.1491; **R*f*** (50% EtOAc/ pet. ether 40-60) = 0.52

(*E*)-2-(1-(methoxymethoxy)-3,7-dimethylocta-2,6-dien-1-yl)-1-methylquinolin-4(1*H*)-one (16)

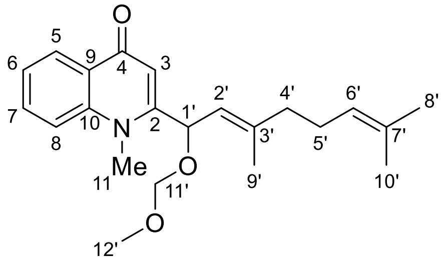

Using the conditions of Wolfe et al.[2] A mixture of **15** (100 mg, 0.23 mmol), Cs_2CO_3 (150 mg, 0.45 mmol), Pd_2dba_3 (10 mg, 0.01 mmol), P(2-furyl)$_3$ (10 mg, 0.045 mmol) and toluene (2.5 mL) was heated and stirred in a sealed tube at 100 °C for 24 h. The mixture was filtered through celite, rinsing with EtOAc (10 mL), and concentrated *in vacuo*. The crude product was purified using flash column chromatography (SiO_2, 60-70% EtOAc/ pet. ether 40-60) to give the title compound as a brown oil (80 mg, 0.23 mmol, 100%).

^{1}H NMR (400 MHz, $CDCl_3$): δ = 8.48 (1H, dd, *J* = 8.2, 1.7 Hz, H-5), 7.71 (1H, ddd, *J* = 8.5, 7.2, 1.7 Hz, H-7), 7.55 (1H, d, *J* = 8.5 Hz, H-8), 7.41 (1H, br t, *J* = 7.5 Hz), 6.57 (1H, s, H-3), 5.46 (1H, d, *J* = 8.5 Hz, H-1’), 5.37 (1H, br d, *J* = 8.9 Hz, H-2’), 5.07-5.02 (1H, m, H-6’), 4.75 (1H, d, *J* = 6.8 Hz, H-11’[a]), 4.68 (1H, d, *J* = 6.8 Hz, H-11’[a]), 3.81 (3H, s, H-11), 3.43 (3H, s, H-12’), 2.16-2.12 (4H, m, H-4’ & H-5’), 1.83 (3H, s, H-9’), 1.67-1.65 (6H, m, H-8’ & H-10’); **^{13}C NMR** (100 MHz, $CDCl_3$): δ = 178.4 (C-4), 152.9 (C-2), 143.8 (C-3’), 142.4 (C-10), 132.3 (C-7 or C-7’), 132.2 (C-7 or C-7’), 126.7 (C-5), 126.6 (C-9), 123.5 (C-6 or C-6’), 123.4 (C-6 or C-6’), 121.3 (C-2’), 115.4 (C-8), 110.6 (C-3), 93.8 (C-11’), 72.2 (C-1’), 56.0 (C-12’), 39.7 (C-6’), 34.9 (C-11), 26.1 (C-5’), 25.7 (C-8’), 17.7 (C-10’), 16.9 (C-9’); ***v*** [cm^{-1}] (neat): 1622 (s, C=O quinolone), 1600 (s, C=C Ar); **HRMS:** *m/z* (ES) calcd for $C_{22}H_{30}NO_3$ $[M+H]^+$: 356.2226, found 356.2240; **R*f*** (70% EtOAc/ pet. ether 40-60) = 0.25

(*E*)-2-(1-hydroxy-3,7-dimethylocta-2,6-dien-1-yl)-1-methylquinolin-4(1*H*)-one (5) & (*E*)-2-(3-hydroxy-3,7-dimethylocta-1,6-dien-1-yl)-1-methylquinolin-4(1*H*)-one (6)

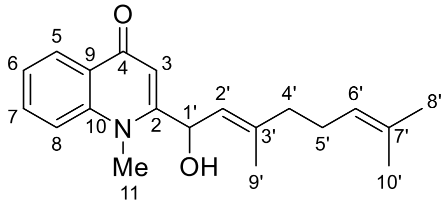

5

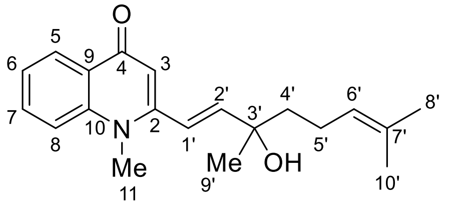

6

A mixture of **16** (62 mg, 0.17 mmol) and pyridinium tosylate (437 mg, 1.74 mmol) in *t*-BuOH (3 mL) was stirred at reflux for 48 h. Following cooling, the mixture was diluted with sat. aq. $NaHCO_3$ (3 mL)

and extracted with EtOAc (3 x 2 mL). The combined organic phases were dried ($MgSO_4$) and concentrated *in vacuo*. Purification by flash column chromatography (SiO_2, 40-100% EtOAc/ Pet. ether 40-60) gave **5** and **6** as 4:1 mixture respectively (based on ^{1}H NMR analysis) (39 mg, 0.13 mmol, 72%). The products were separated by semi-preparative HPLC (5-95% B) to provide **5** and **6** as off-white semi-solids.

*(E)-2-(1-hydroxy-3,7-dimethylocta-2,6-dien-1-yl)-1-methylquinolin-4(1H)-one (**5**):*

^{1}H NMR (500 MHz, $CDCl_3$): δ = 8.27 (1H, dd, J = 7.9, 1.5 Hz, H-5), 7.50 (1H, ddd, J = 8.9, 7.0, 1.8 Hz, H-7) 7.33 (1H, ddd, J = 7.6, 7.0, 0.6 Hz, H-6), 7.19 (1H, d, J = 8.6 Hz, H-8), 6.45 (1H, s, H-3), 5.48-5.42 (2H, m, H-1' & H-2'), 5.06-5.02 (1H, m, H-6'), 3.70 (3H, s, H-11), 2.12-2.05 (4H, m, H-4' & H-5'), 1.72 (3H, d, J = 0.9 Hz, H-9'), 1.65 (3H, s, H-8'), 1.56 (3H, s, H-10'); **^{13}C NMR** (125 MHz, $CDCl_3$): δ = 178.2 (C-4), 156.0 (C-2), 141.9 (C-10 or C-3'), 141.8 (C-3' or C-10'), 132.1 (C-7), 132.0 (C-7'), 126.0 (C-5), 125.5 (C-9), 123.6 (C-6'), 123.3 (C-2'), 123.2 (C-6), 115.3 (C-8), 110.7 (C-3), 69.8 (C-1'), 39.6 (C-4'), 35.0 (C-11), 26.2 (C-5'), 25.7 (C-8'), 17.7 (C-10'), 17.0 (C-9'); ***ν*** [cm^{-1}] (neat): 3240 (br, O-H), 1618 (m, C=O quinolone), 1597 (s, C=C Ar), 1556 (m, C=C Ar), 1499 (m, C=C Ar), 1467 (m, C=C Ar), 1443 (m, C=C Ar); **HPLC** t_r = 11.04 mins (5-95% B); analytical data consistent with that previously published (slight changes in the NMR data were observed on a sample by sample basis, likely due to minute variations in the amount of water present in the samples. Note that H-8', H-9' and H-10' are reassigned relative to the original isolation paper, on the basis of 2D NMR data).[3]

*(E)-2-(3-hydroxy-3,7-dimethylocta-1,6-dien-1-yl)-1-methylquinolin-4(1H)-one (**6**):*

^{1}H NMR (400 MHz, $CDCl_3$): δ = 8.42 (1H, dd, J = 8.0, 1.6 Hz, H-5), 7.64 (1H, ddd, J = 8.4, 6.8, 1.6 Hz, H-7), 7.44 (1H, d, J = 8.8 Hz, H-8), 7.36 (1H, t, J = 7.4 Hz, H-6), 6.72 (1H, d, J = 15.6 Hz, H-1'), 6.35 (1H, d, J = 14.8 Hz, H-2'), 6.34 (1H, s, H-3), 5.12 (1H, m, H-6'), 3.69 (3H, s, H-11), 2.09 (2H, m, H-5'), 1.70 (2H, m, H-4'), 1.68 (3H, s, H-8'), 1.60 (3H, s, H-10'), 1.39 (3H, s, H-9'); **^{13}C NMR** (125 MHz, $CDCl_3$): δ = 177.9 (C-4), 152.3 (C-2), 147.0 (C-13), 141.5 (C-9), 132.5 (C-10), 132.2 (C-7), 126.7 (C-18), 126.6 (C-5), 123.9 (C-17), 123.4 (C-6), 121.6 (C-12), 115.4 (C-8), 109.7 (C-3), 73.4 (C-14), 42.1 (C-15), 35.5 (C-11), 28.3 (C-20), 25.7 (C-21), 22.9 (C-16), 17.8 (C-19); ***ν*** [cm^{-1}] (neat): 3242 (br, O-H), 1655 (w, C=C), 1618 (C=O), 1596 (s, quinolone ring), 1552 (s, quinolone ring); **HPLC** t_r = 10.11 mins (5-95% B); analytical data consistent with that previously published.[3]

(*E*)-2-(3,7-dimethylocta-2,6-dien-1-yl)-3-methylquinolin-4(1*H*)-one (1)

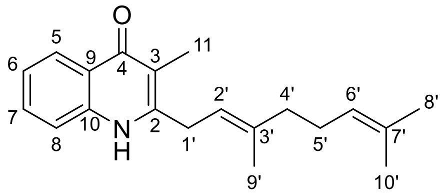

To a solution of 2-(chloromethyl)-3-methylquinolin-4(1*H*)-one (prepared by our previously published procedure)[4] (263 mg, 1.27 mmol) and (*E*)-2-(2,6-dimethylhepta-2,-5-dien-1-yl)-4,4,5,5-tetramethyl-1,3-2-dioxaborolane (prepared by our previously published procedure)[4] (264 mg, 0.63 mmol) in 1,4-dioxane (5 mL) was added a solution of sodium carbonate (2 M, 5 mL) and $Pd(PPh_3)_4$ (139 g, 0.12 mmol). The reaction vessel was sealed followed by heating at 120 °C for 2 h. The mixture was filtered through celite, rinsing with EtOAc. The organic phase was washed with brine (2 x 10 mL), dried over $MgSO_4$ and concentrated *in vacuo*. The crude material was purified by flash column chromatography (SiO_2, 50% EtOAc) to obtain the desired compound as an off-white solid (141 mg, 0.48 mmol, 38%)

^{1}H NMR (400 MHz, $CDCl_3$): δ = 9.82 (1H, br s, N-H), 8.36 (1H, d, *J* = 7.8 Hz, H-5), 7.50 (1H, t, *J* = 7.7 Hz, H-7), 7.40 (1H, d, *J* = 8.2 Hz, H-8), 7.25 (1H, t, *J* = 7.5 Hz, H-6), 5.29 (1H, t, *J* = 7.0 Hz, H-2'), 5.10-5.04 (1H, m, H-6'), 3.50 (2H, d, *J* = 7.2 Hz, H-1'), 2.15 (3H, s, H-10), 2.14-2.08 (4H, m, H-4' & H-5'), 1.67 (6H, s, H-8' & H-9'), 1.59 (3H, s, H-10'); **^{13}C NMR** (100 MHz, $CDCl_3$): δ = 177.9 (C-4), 147.7 (C-2), 141.6 (C-10), 138.8 (C-3'), 132.2 (C-7'), 131.0 (C-7), 126.1 (C-5), 123.8 (C-9), 123.6 (C-6'), 123.1 (C-6), 117.4 (C-8), 117.3 (C-2'), 115.5 (C-3), 39.6 (C-4'), 31.2 (C-1'), 26.4 (C-5'), 25.8 (C-8'), 17.8 (C-10'), 16.4 (C-9'), 10.5 (C-11); ***ν*** [cm^{-1}] (neat): 1637 (m, C=O quinolone), 1607 (w, C=C Ar), 1589 (m, C=C Ar), 1549 (m, C=C Ar), 1492 (s, C=C Ar), 1472 (s, C=C Ar), 1444 (m, C=C Ar), 1357 (s, C=C Ar); **mp** (EtOAc): 188-189 °C (lit. value: 199-200 °C); analytical data consistent with that previously published (slight changes in the NMR data were observed on a sample by sample basis, likely due to minute variations in the amount of water present in the samples).[3]

(*E*)-2-(3,7-dimethylocta-2,6-dien-1-yl)-1,3-dimethylquinolin-4(1*H*)-one (4)

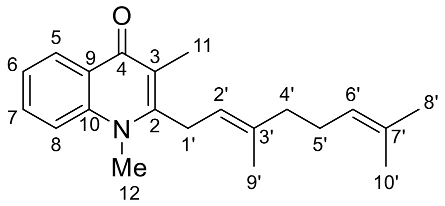

To a suspension of **1** (25 mg, 0.085 mmol) in THF (0.5 mL) was added LiO*t*-Bu (14 mg, 0.17 mmol), followed by stirring at RT for 20 min. Iodomethane (21 µL, 0.34 mmol) was added at 0 °C followed by stirring at RT for 3 h, at which point LCMS indicated incomplete reaction so a further portion of iodomethane (21 µL, 0.34 mmol) followed by stirring at RT for 12 h. Sat. aq. $NaHCO_3$ (2 mL) was added, followed by extraction with EtOAc (3 x 2 mL). The combined organic extracts were dried ($MgSO_4$) and concentrated *in vacuo*. The crude product was purified using flash column

chromatography (SiO_2, 40% EtOAc/ pet. ether 40-60) to give the title compound as an off-white semi-solid (16 mg, 0.051 mmol, 60%)

^{1}H NMR (500 MHz, $CDCl_3$): δ = 8.48 (1H, dd, J = 8.2, 1.8 Hz, H-5), 7.61 (1H, ddd, J = 8.9 Hz, 7.0, 1.8 Hz, H-7), 7.46 (1H, d, J = 8.5 Hz, H-8) 7.33 (1H, ddd, J = 7.9, 6.7, 0.6 Hz, H-6), 5.08-5.01 (1H, m, H-2'), 3.72 (3H, s, H-11), 3.56 (1H, dd, J = 6.1, 0.9 Hz, H-1'), 2.22 (3H, s, H-12), 2.13-2.04 (4H, m, H-4' & H-5'), 1.78 (3H, d, J = 1.2 Hz, H-9'), 1.65 (3H, d, J = 0.9 Hz, H-8'), 1.58 (3H, s, 10'); **^{13}C NMR** (125 MHz, $CDCl_3$): δ = 177.1 (C-4), 150.6 (C-2), 141.1 (C-10), 139.0 (C-3'), 131.9 (C-7'), 131.5 (C-7), 127.0 (C-5), 124.9 (C-9), 123.5 (C-6'), 122.7 (C-6), 118.3 (C-2'), 117.6 (C-3), 115.0 (C-8), 39.4 (C-4'), 34.8 (C-12), 31.0 (C-1'), 26.5 (C-5'), 25.7 (C-8'), 17.7 (C-10'), 16.5 (C-9'), 11.6 (C-11); ***ν*** [cm^{-1}] (neat): 1614 (m, C=O quinolone), 1593 (s, C=C Ar), 1540 (s, C=C Ar), 1499 (m, C=C Ar), 1471 (m, C=C Ar), 1436 (m, C=C Ar); analytical data consistent with that previously published.[3]

(*E*)-2-(5-(3,3-dimethyloxiran-2-yl)-3-methylpent-2-en-1-yl)-1,3-dimethylquinolin-4(1*H*)-one (8)

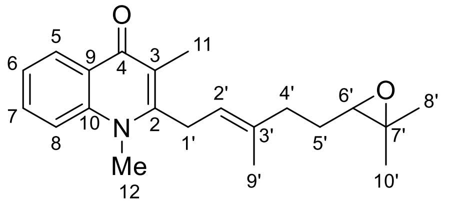

To a solution of **4** (23 mg, 0.074 mmol) in CH_2Cl_2 (0.5 mL) was added $NaHCO_3$ (8 mg, 0.096 mmol). The mixture was cooled to 0 °C and *m*-CPBA (77% purity, 18 mg, 0.081 mmol) was added, followed by stirring at RT for 30 min. H_2O (1 mL) was added and the phases separated. The organic phase was watched with sat. aq. $NaHCO_3$ (0.5 mL), and then the combined aqueous phases were extracted with EtOAc (2 x 2 mL). The combined organic phases were dried (Na_2SO_4) and concentrated *in vacuo*. The crude product was purified using flash column chromatography (SiO_2, 70-80% EtOAc/ pet. ether 40-60) to give the title compound as a colourless semi-solid (10.3 mg, 0.0316 mmol, 43%).

^{1}H NMR (500 MHz, $CDCl_3$): δ = 8.48 (1H, dd, J = 7.9, 1.5 Hz, H-5), 7.62 (1H, ddd, J = 8.9, 7.0, 1.8 Hz, H-7), 7.47 (1H, d, J = 8.5 Hz, H-8) 7.33 (1H, ddd, J = 7.9, 7.0, 0.9 Hz, H-6), 5.15-5.11 (1H, m, H-2'), 3.73 (3H, s, H-12), 3.59 (2H, d, J = 6.1 Hz, H-1'), 2.67 (1H, dd, J = 7.0, 5.2 Hz, H-6'), 2.29-2.14 (2H, m, H-4'), 2.22 (3H, s, H-11), 1.82 (3H, d, J = 1.2 Hz, H-9'), 1.72-1.60 (2H, m, H-5'), 1.28 (3H, s, H-8'), 1.25 (3H, s, H-10'); **^{13}C NMR** (125 MHz, $CDCl_3$): δ = 177.1 (C-4), 150.3 (C-2), 141.1 (C-10), 138.4 (C-3'), 131.5 (C-7), 127.0 (C-5), 124.9 (C-9), 122.8 (C-6), 118.7 (C-2'), 117.4 (C-3), 115.0 (C-8), 63.9 (C-6'), 58.2 (C-7'), 36.4 (C-4'), 35.0 (C-12), 30.6 (C-1'), 27.4 (C-5'), 24.8 (C-8'), 18.8 (C-10'), 16.6 (C-9'), 11.7 (C-11); ***ν*** [cm^{-1}] (neat): 1614 (m, C=O quinolone), 1597 (s, C=C Ar), 1571 (m, C=C Ar), 1540 (s, C=C Ar), 1500 (m, C=C Ar), 1471 (m, C=C Ar); analytical data consistent with that previously published.[3]

(*E*)-2-(3,7-dimethylocta-2,6-dien-1-yl)-3-methyl-1-((methylthio)methyl)quinolin-4(1*H*)-one (7)

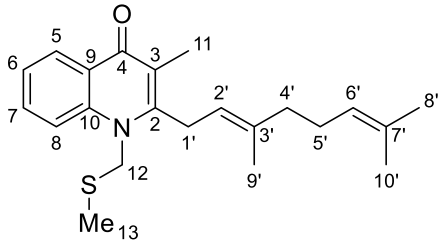

To a suspension of **1** (52 mg, 0.18 mmol) in THF (0.5 mL) was added LiO*t*-Bu (1M solution in THF, 0.2 mL, 0.2 mmol). The resulting solution was stirred for 20 m at RT, followed by addition of chloromethyl methyl sulfide (55 µL, 3.7 mmol) at 0 °C. The mixture was warmed to RT, followed by stirring for 12 h. Water (2 mL) was added, followed by extraction with EtOAc (3 x 1 mL). The combined organic phases were dried (Na_2SO_4) and concentrated *in vacuo*. The crude mixture was separated using flash column chromatography (SiO_2, 30-50 % EtOAc/ Pet. ether 40-60) to give recovered starting material (20 mg, 0.068 mmol, 37%) and the title compound as a yellow semi-solid (3.8 mg, 0.11 mmol, 6%).

^{1}H NMR (500 MHz, $CDCl_3$): δ = 8.47 (1H, dd, J = 7.9, 1.5 Hz, H-5), 7.67-7.60 (2H, m, H-7 & H-8), 7.35 (1H, ddd, J = 7.9, 6.4, 1.5 Hz, H-6), 5.14 (2H, s, H-12), 5.12-5.07 (1H, m, H-2'), 5.07-5.02 (1H, m, H-6'), 3.70 (2H, d, J = 6.1 Hz, H-1'), 2.22 (6H, m, H-11 & H-13), 2.14-2.07 (4H, m, H-4' & H-5'), 1.83 (3H, d, J = 0.9 Hz, H-8'), 1.67 (3H, s, H-10'), 1.60 (3H, s, H-9' [overlaps with water peak]); **^{13}C NMR** (125 MHz, $CDCl_3$): δ = 177.4 (C-4), 149.9 (C-2), 140.3 (C-10), 139.5 (C-3'), 131.9 (C-7'), 131.6 (C-7), 127.2 (C-5), 124.9 (C-9), 123.6 (C-6), 123.2 (C-6'), 118.6 (C-2'), 118.2 (C-3), 115.6 (C-8), 49.6 (C-12), 39.4 (C-4'), 30.1 (C-1'), 26.3 (C-5'), 25.7 (C-8'), 17.7 (C-10'), 16.5 (C-9'), 14.5 (C-13), 11.5 (C-11); ***v*** [cm^{-1}] (neat): 1615 (m, C=O quinolone), 1597 (s, C=C Ar), 1545 (m, C=C Ar), 1491 (m, C=C Ar); analytical data consistent with that previously published.[3]

References

[1] M. Lautens, M. L. Maddess, *Org. Lett.* **2004**, *6*, 1883–6.

[2] J. P. Wolfe, R. A. Rennels, S. L. Buchwald, *Tetrahedron* **1996**, *52*, 7525–7546.

[3] K. A. Dekker, T. Inagaki, T. D. Gootz, L. H. Huang, Y. Kojima, W. E. Kohlbrenner, Y. Matsunaga, P. R. McGuirk, E. Nomura, T. Sakakibara, et al., *J. Antibiot. (Tokyo).* **1998**, *51*, 145–152.

[4] F. Salvaggio, J. T. Hodgkinson, L. Carro, S. M. Geddis, W. R. J. D. Galloway, M. Welch, D. R. Spring, *European J. Org. Chem.* **2016**, *2016*, 434–437.

^{1}H and ^{13}C spectra

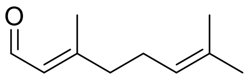

10

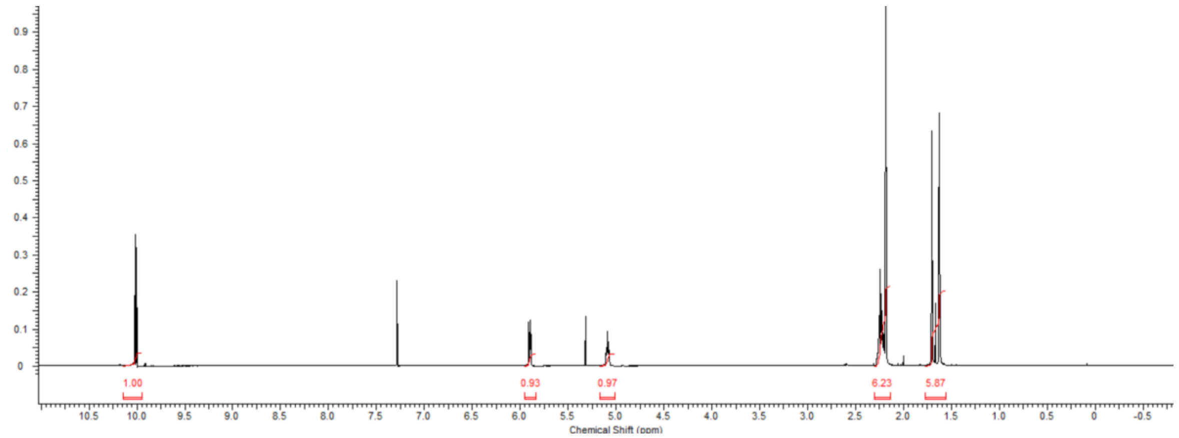

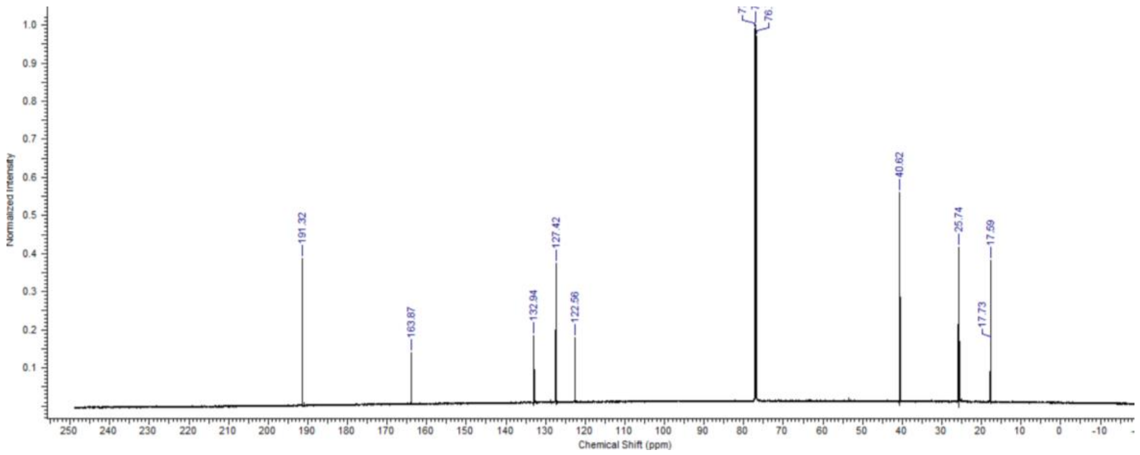

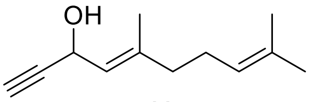
OH

11

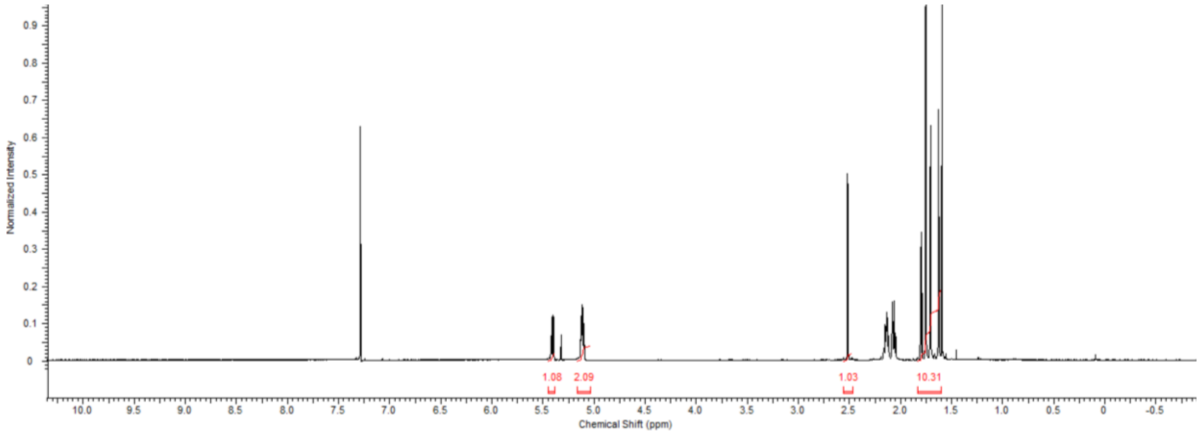
Normalized Intensity
Chemical Shift (ppm)
1.08
2.09
1.03
10.31

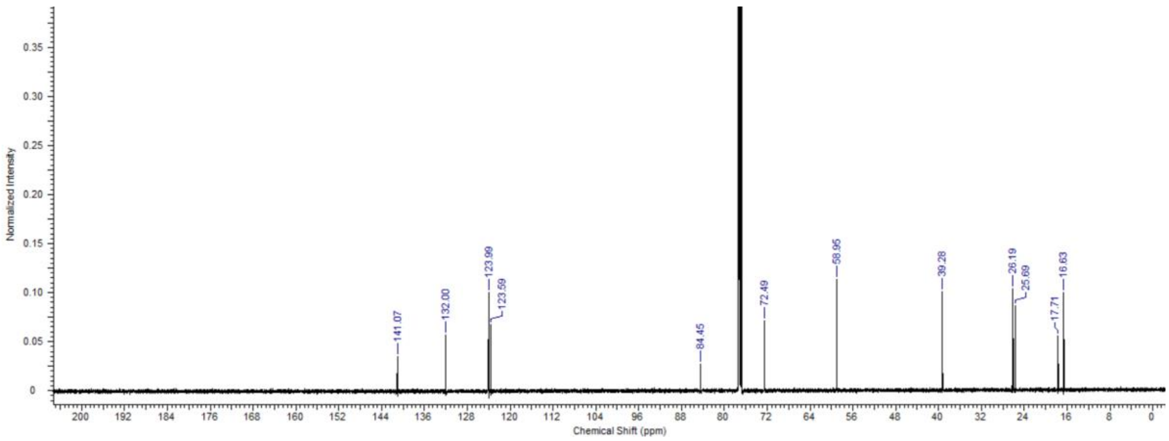
Normalized Intensity
Chemical Shift (ppm)
141.07
132.00
123.99
123.59
84.45
72.49
58.95
39.28
26.19
25.69
17.71
16.63

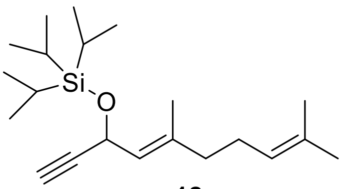
Si
O

12

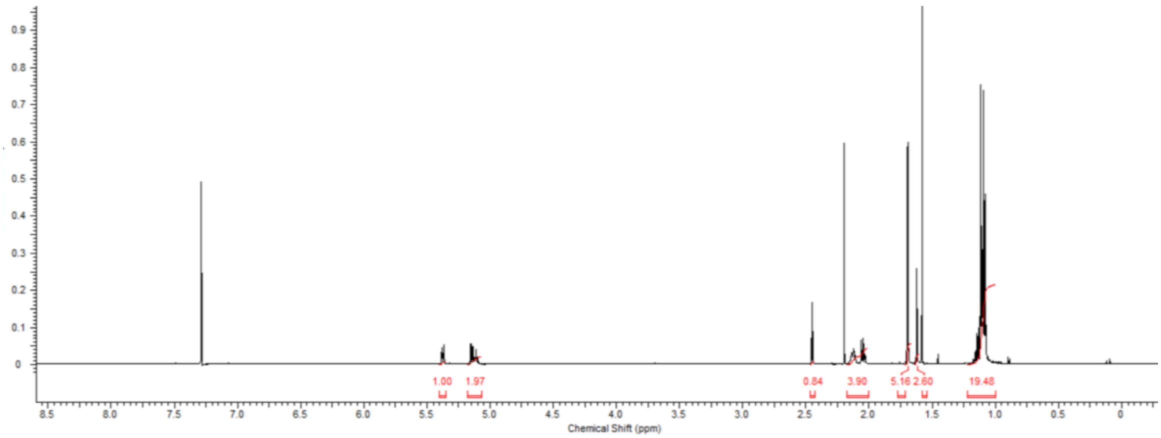
1.00
1.97
0.84
3.90
5.16
2.60
19.48
Chemical Shift (ppm)

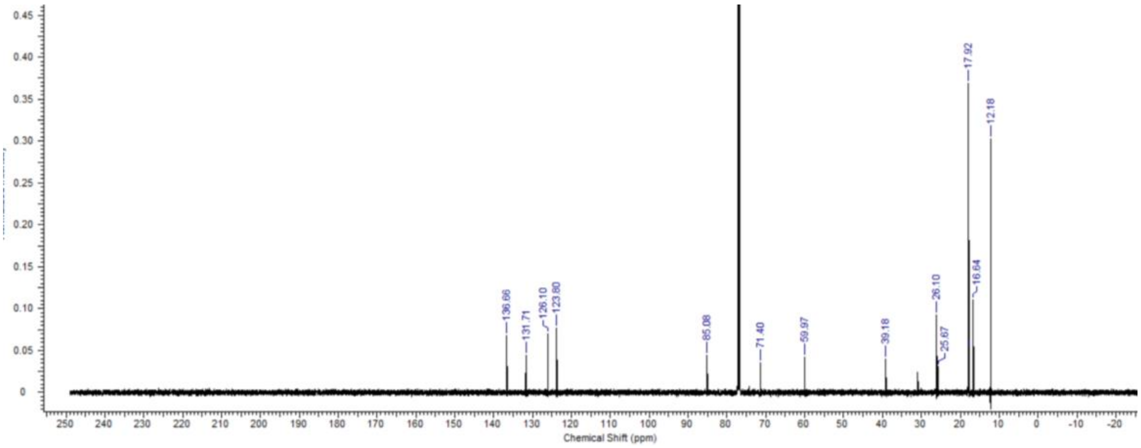
136.66
131.71
126.10
123.80
85.08
71.40
59.97
39.18
26.10
25.67
17.92
16.64
12.18
Chemical Shift (ppm)

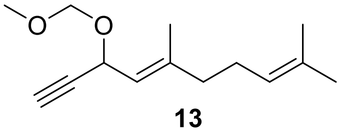

13

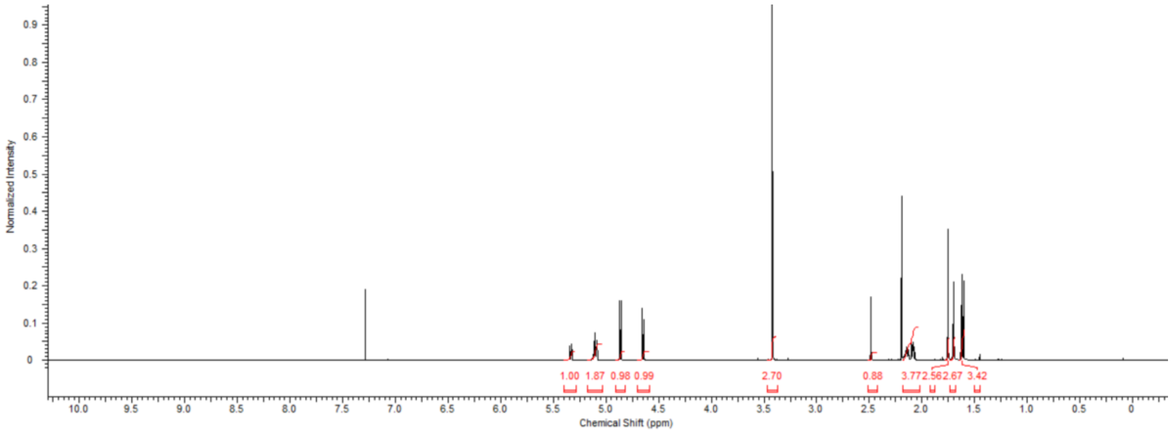

Normalized Intensity
1.00 1.87 0.98 0.99 2.70 0.88 3.77 2.56 2.67 3.42
Chemical Shift (ppm)

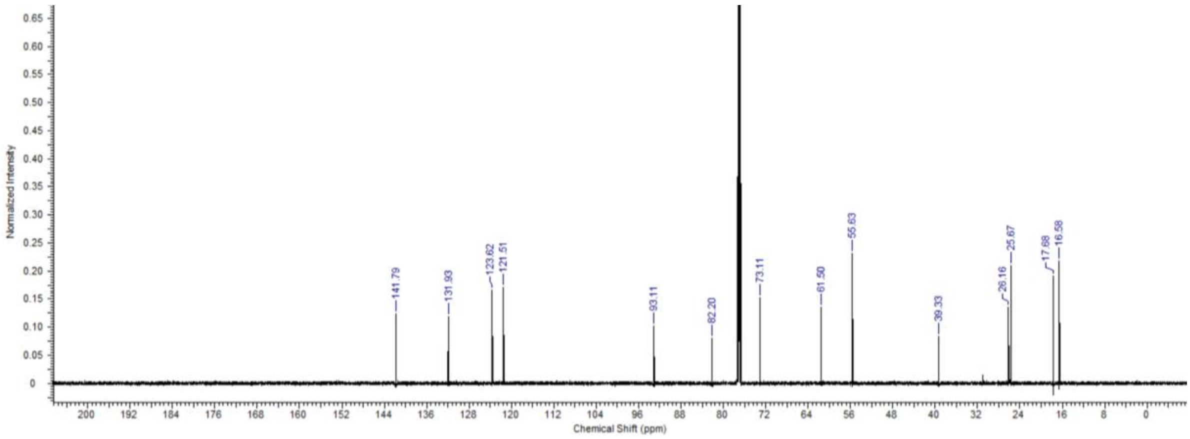

Normalized Intensity
141.79
131.93
123.62
121.51
93.11
82.20
73.11
61.50
55.63
39.33
26.16
25.67
17.68
16.58
Chemical Shift (ppm)

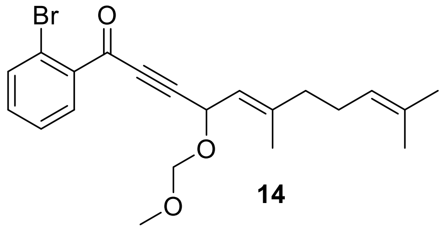
Br
O
O
O

14

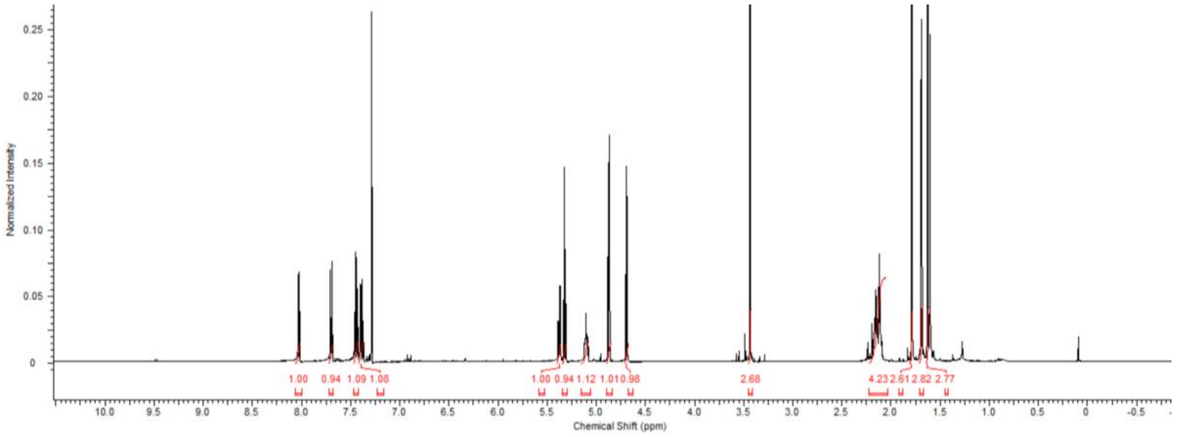
Normalized Intensity
1.00
0.94
1.09
1.08
1.00
0.94
1.12
1.01
0.98
2.68
4.23
2.61
2.82
2.77
Chemical Shift (ppm)

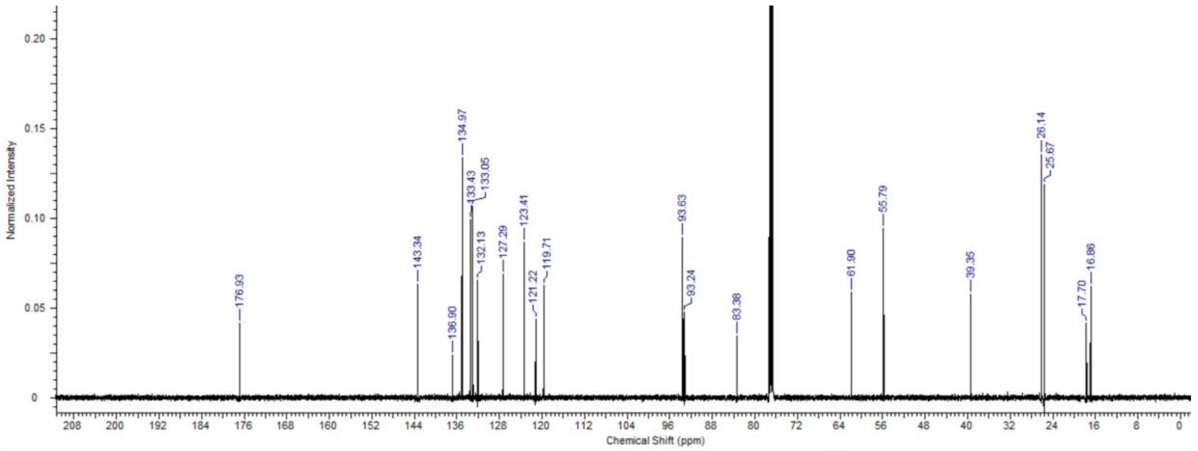
Normalized Intensity
176.93
143.34
136.90
134.97
133.43
133.05
132.13
127.29
123.41
121.22
119.71
93.63
93.24
83.38
61.90
55.79
39.35
26.14
25.67
17.70
16.86
Chemical Shift (ppm)

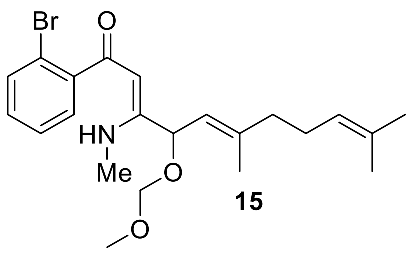
Br
O
HN
Me
O
O

15

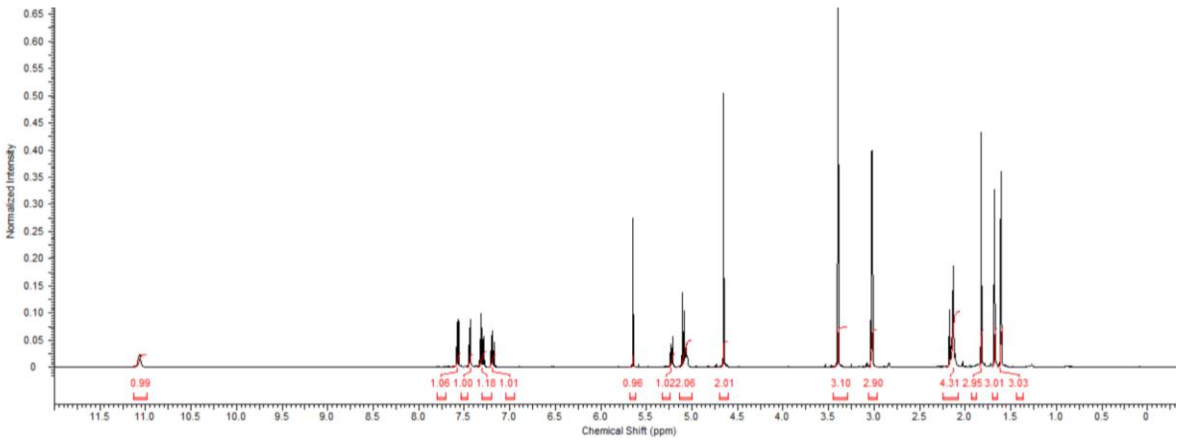
Normalized Intensity
Chemical Shift (ppm)
0.99
1.06
1.00
1.18
1.01
0.96
1.02
2.06
2.01
3.10
2.90
4.31
2.95
3.01
3.03

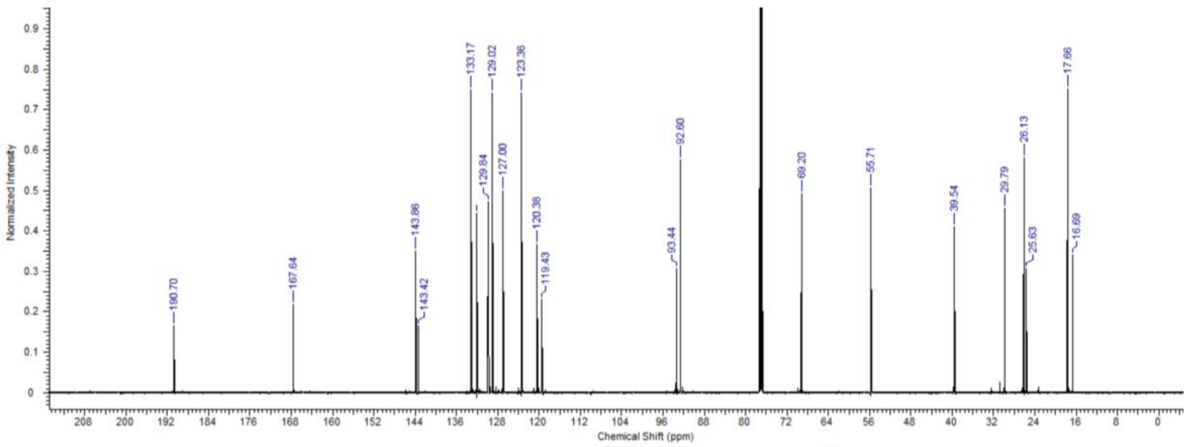
Normalized Intensity
Chemical Shift (ppm)
190.70
167.64
143.86
143.42
133.17
129.84
129.02
127.00
123.36
120.38
119.43
93.44
92.60
69.20
55.71
39.54
29.79
26.13
25.63
17.66
16.69

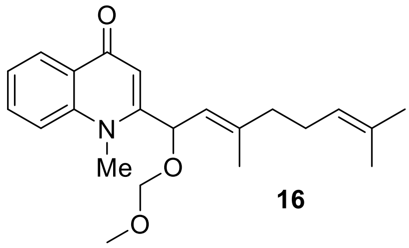
O
N
Me
O
O

16

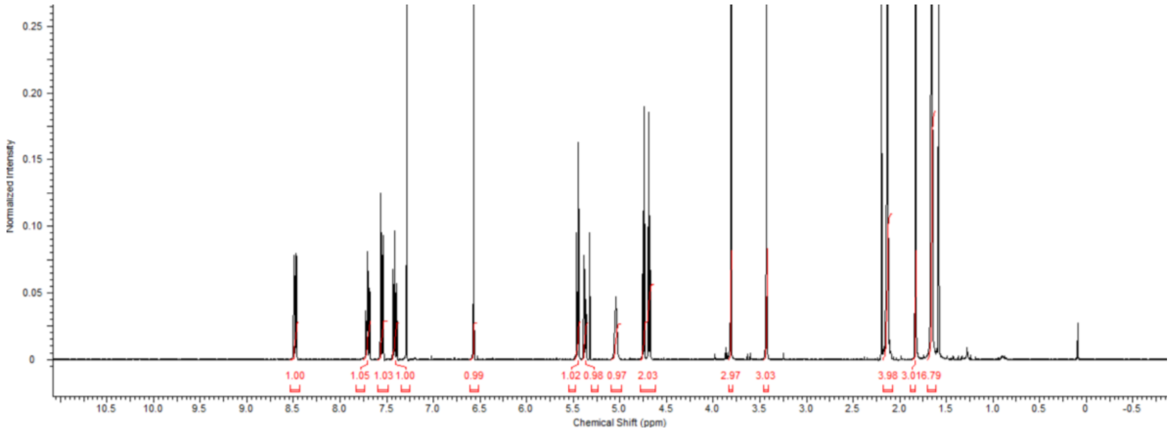
Normalized Intensity
Chemical Shift (ppm)
1.00
1.05
1.03
1.00
0.99
1.02
0.98
0.97
2.03
2.97
3.03
3.98
3.01
6.79

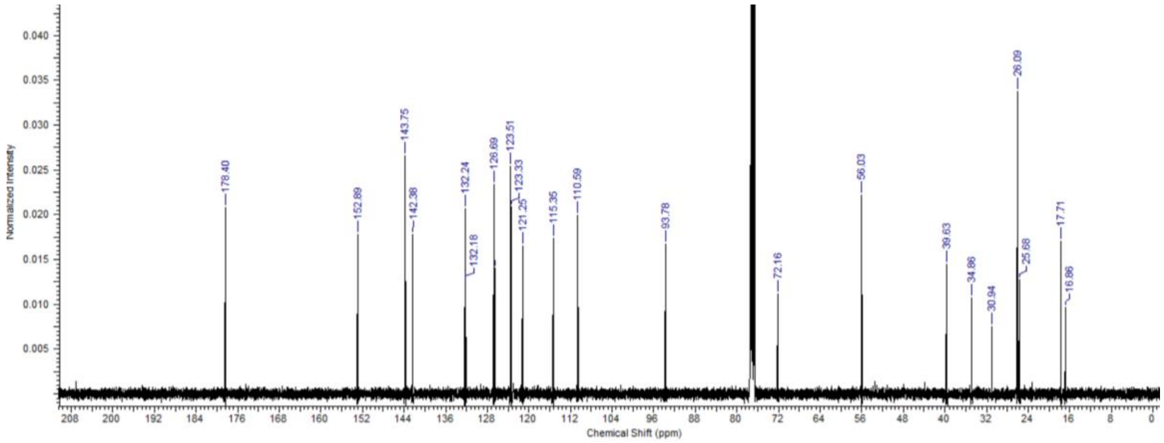
Normalized Intensity
Chemical Shift (ppm)
178.40
152.89
143.75
142.38
132.24
132.18
126.69
123.51
123.33
121.25
115.35
110.59
93.78
72.16
56.03
39.63
34.86
30.94
26.09
25.68
17.71
16.86

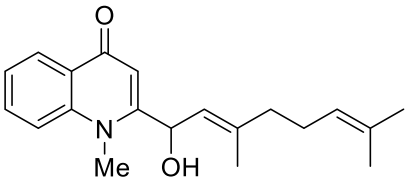
O
N
Me
OH

5

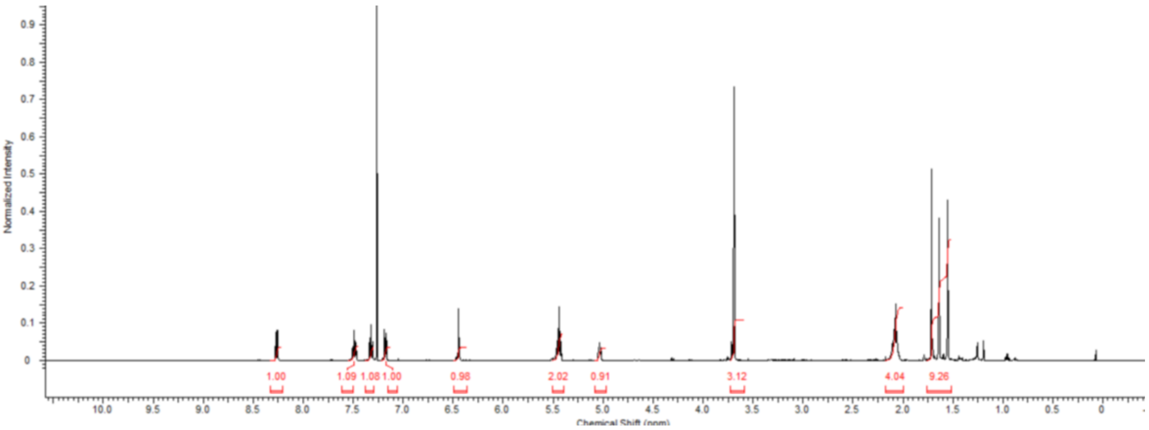
Normalized Intensity
1.00
1.09
1.08
1.00
0.98
2.02
0.91
3.12
4.04
9.26
Chemical Shift (ppm)

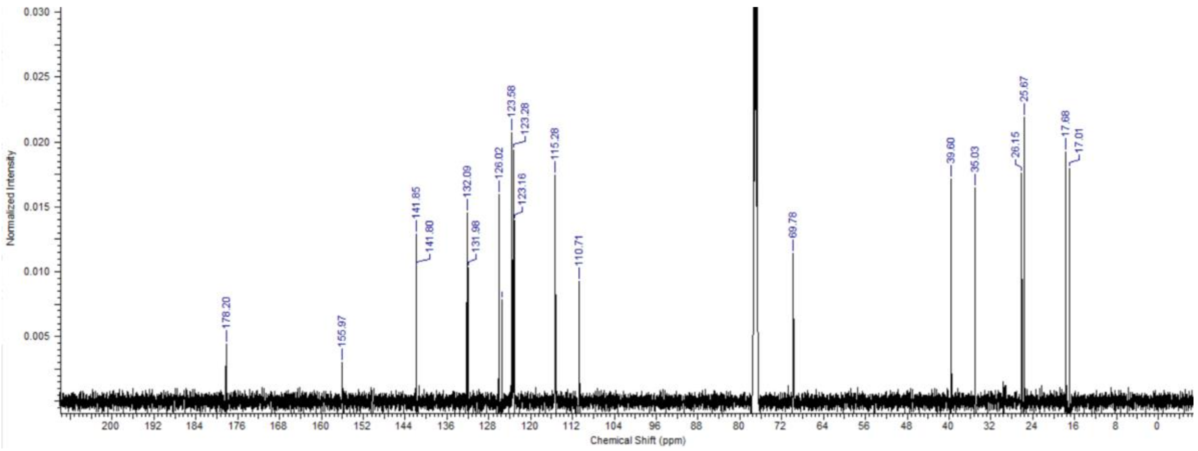
Normalized Intensity
178.20
155.97
141.85
141.80
132.09
131.98
126.02
123.58
123.28
123.16
115.28
110.71
69.78
39.60
35.03
26.15
25.67
17.68
17.01
Chemical Shift (ppm)

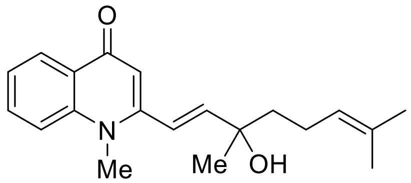
O
N
Me
Me
OH

6

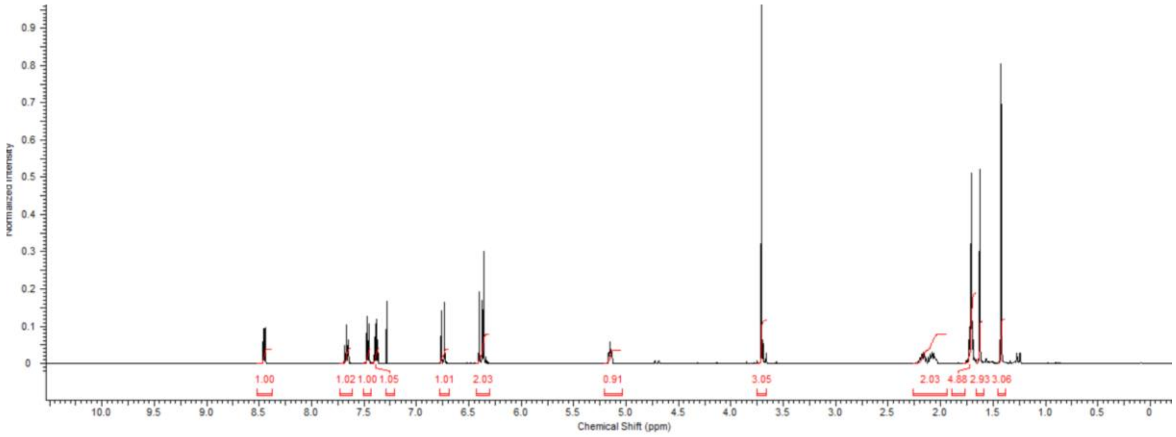
Normalized Intensity
1.00
1.02
1.00
1.05
1.01
2.03
0.91
3.05
2.03
4.88
2.93
3.06
Chemical Shift (ppm)

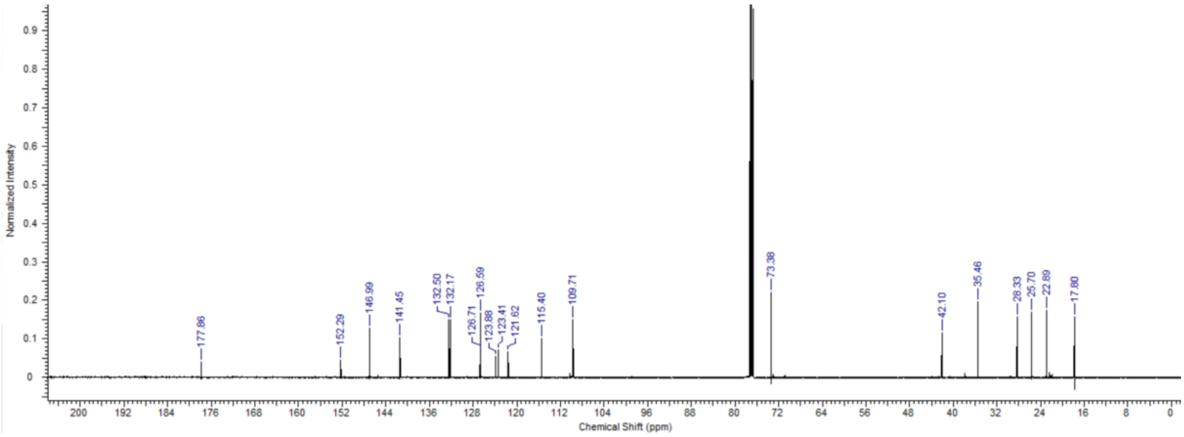
Normalized Intensity
177.86
152.29
146.99
141.45
132.50
132.17
126.71
126.59
123.88
123.41
121.62
115.40
109.71
73.38
42.10
35.46
28.33
25.70
22.89
17.80
Chemical Shift (ppm)

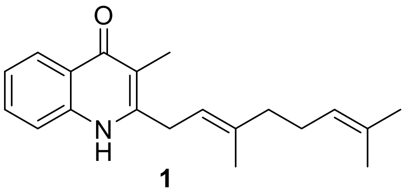
O
N
H

1

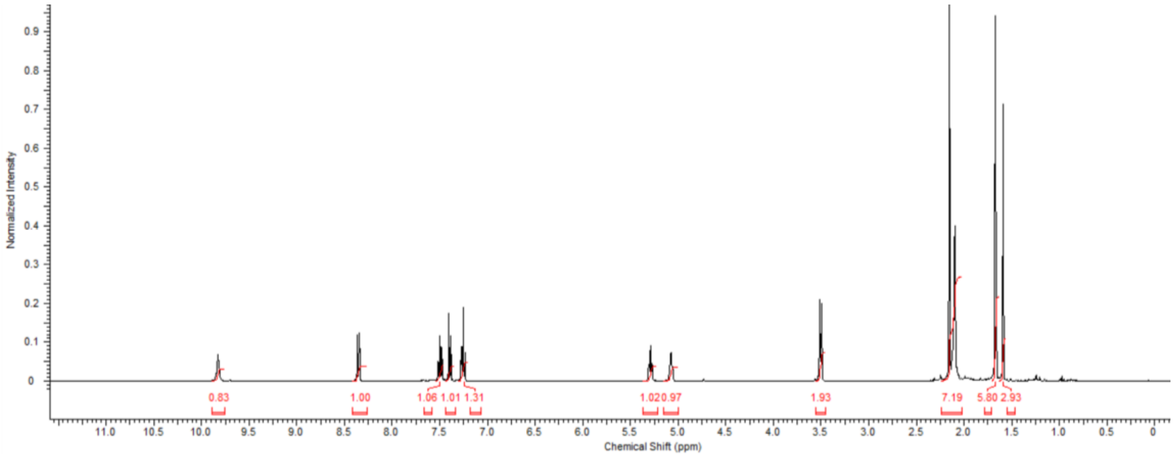
Normalized Intensity
Chemical Shift (ppm)
0.83
1.00
1.06
1.01
1.31
1.02
0.97
1.93
7.19
5.80
2.93

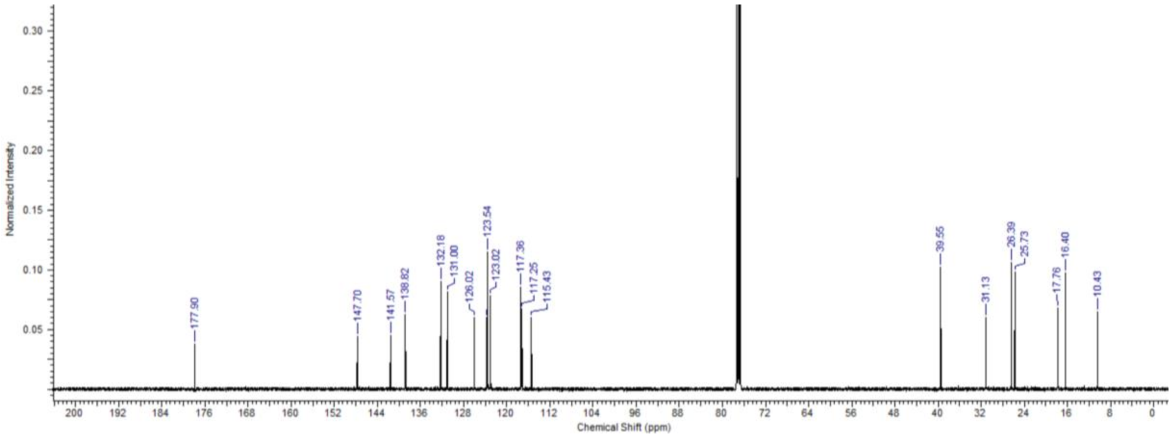
Normalized Intensity
Chemical Shift (ppm)
177.90
147.70
141.57
138.82
132.18
131.00
126.02
123.54
123.02
117.36
117.25
115.43
39.55
31.13
26.39
25.73
17.76
16.40
10.43

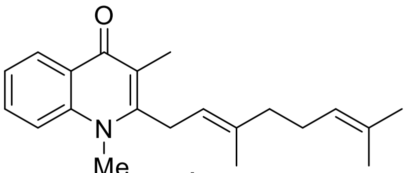
O
N
Me

4

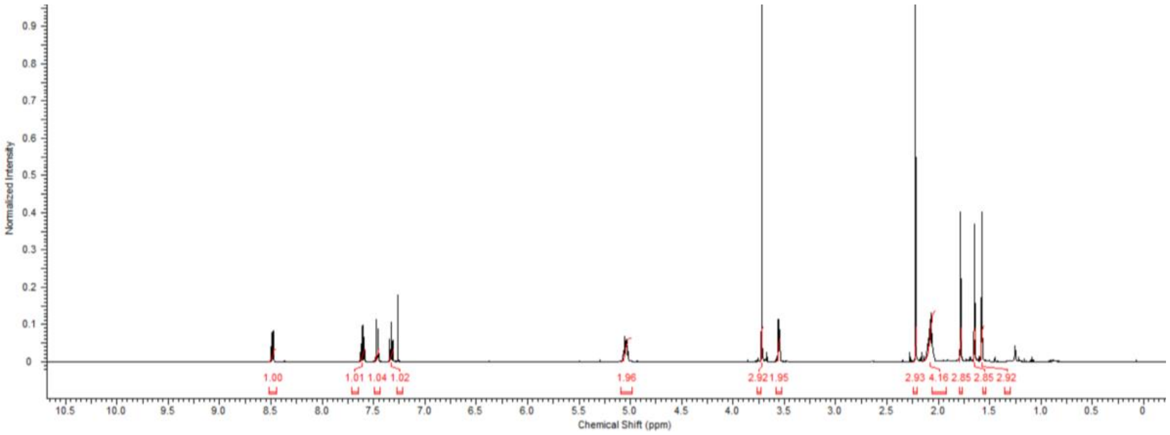
Normalized Intensity
Chemical Shift (ppm)
1.00
1.01
1.04
1.02
1.96
2.92
1.95
2.93
4.16
2.85
2.85
2.92

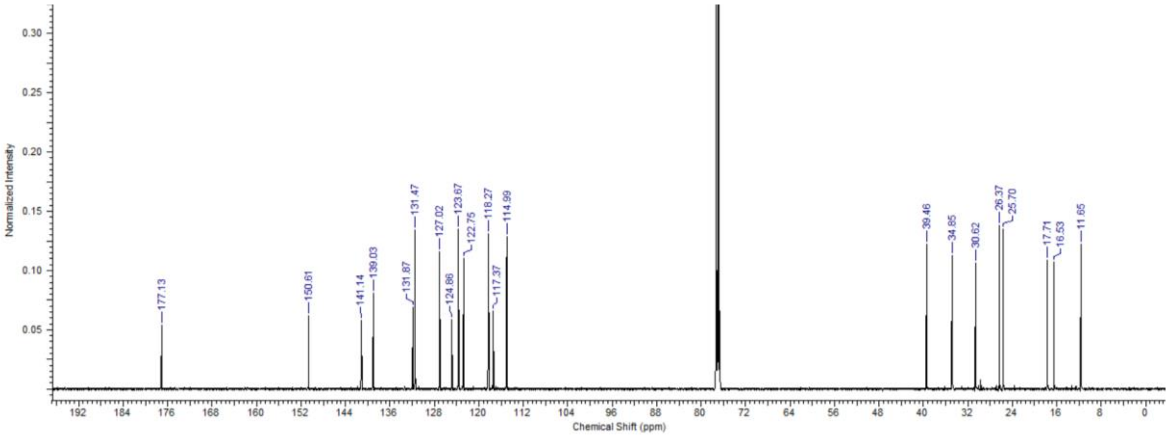
Normalized Intensity
Chemical Shift (ppm)
177.13
150.61
141.14
139.03
131.87
131.47
127.02
124.86
123.67
122.75
118.27
117.37
114.99
39.46
34.85
30.62
26.37
25.70
17.71
16.53
11.65

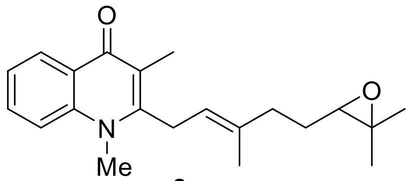

8

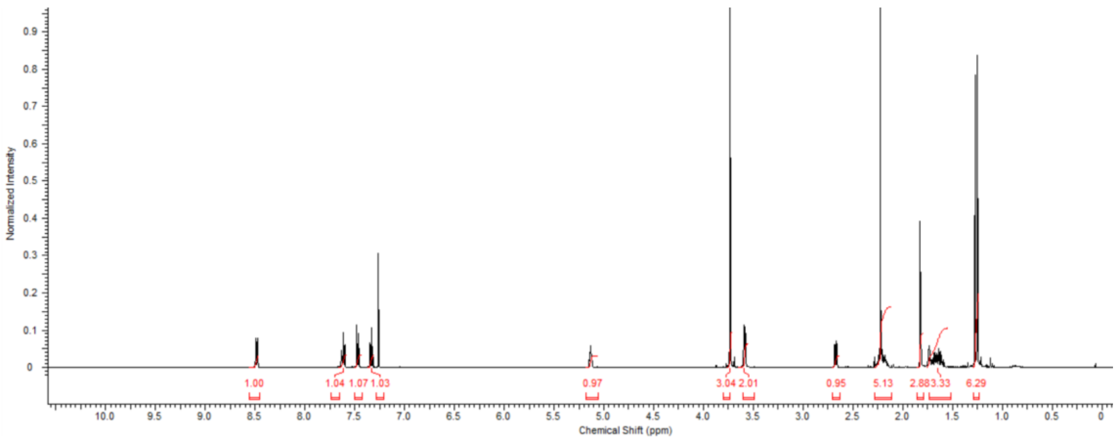
Normalized Intensity
Chemical Shift (ppm)
1.00
1.04
1.07
1.03
0.97
3.04
2.01
0.95
5.13
2.88
3.33
6.29

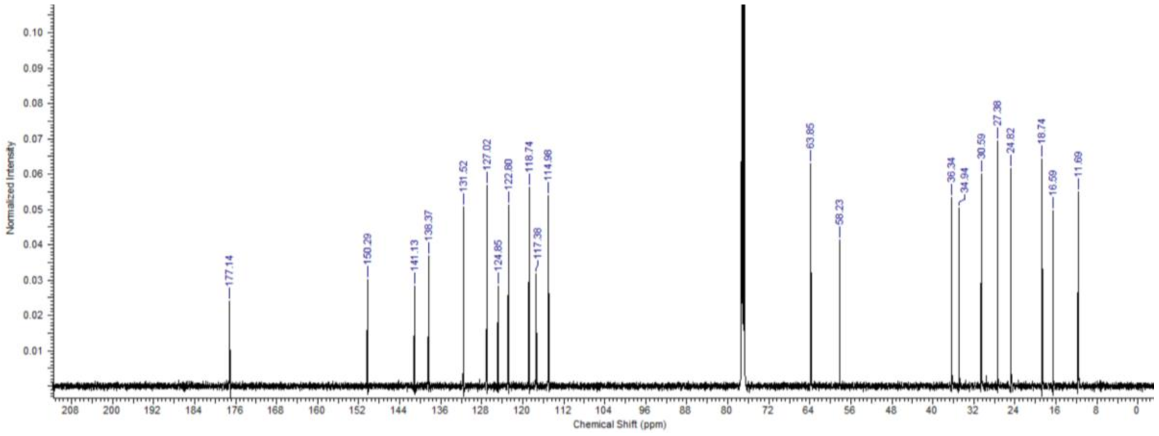
Normalized Intensity
Chemical Shift (ppm)
177.14
150.29
141.13
138.37
131.52
127.02
124.85
122.80
118.74
117.38
114.98
63.85
58.23
36.34
34.94
30.59
27.38
24.82
18.74
16.59
11.69

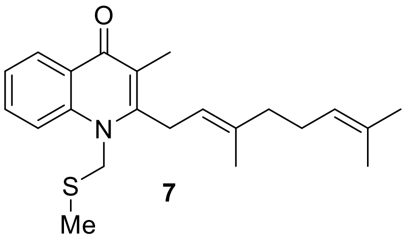

7

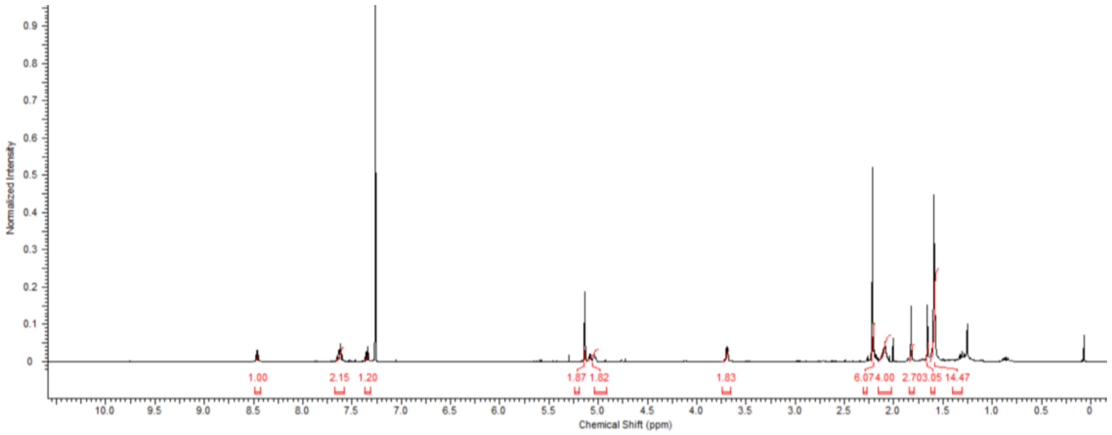

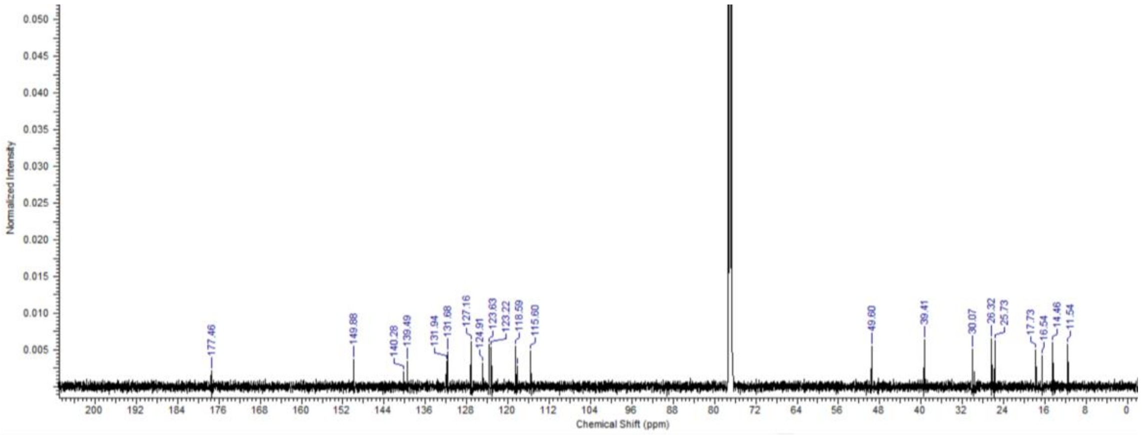

Supplement: Supplementary file 1 — Supporting Information [file EJOC-2016-5799-s001.pdf]
